# Features of cytomegalovirus infection and evaluation of cytomegalovirus-specific T cells therapy in children’s patients following allogeneic hematopoietic stem cell transplantation: A retrospective single-center study

**DOI:** 10.3389/fcimb.2022.1027341

**Published:** 2022-10-20

**Authors:** Yongsheng Ruan, Tingting Luo, Qiujun Liu, Xuan Liu, Libai Chen, Jianyun Wen, Yuhua Xiao, Danfeng Xie, Yuelin He, Xuedong Wu, Xiaoqin Feng

**Affiliations:** Department of Pediatrics, Nanfang Hospital, Southern Medical University, Guangzhou, China

**Keywords:** allogeneic hematopoietic stem cell transplantation, children, cytomegalovirus infection, haploidentical transplant, cytomegalovirus-specific cytotoxic T lymphocytes

## Abstract

Cytomegalovirus (CMV) infection remains a critical cause of mortality after allogeneic hematopoietic stem cell transplantation (allo-HSCT), despite improvement by pre-emptive antivirus treatment. CMV-specific cytotoxic T lymphocytes (CMV-CTL) are universally used and proven well-tolerance after allo-HSCT in adult clinical trials. However, it is not comprehensively evaluated in children’s patients. Herein, we conducted a retrospective study to determine the risk factors of CMV infection and evaluation of CMV-CTL in children patients who underwent allo-HSCT. As result, a significantly poor 5-year overall survival was found in the CMV infection group (87.3 vs. 94.6%, p=0.01). Haploidentical HSCT (haplo-HSCT) was identified as an independent risk factor for CMV infection through both univariate and multivariate analyses (p<0.001, p=0.027, respectively). Furthermore, the cumulative incidence of CMV infection was statistically higher in the haplo-HSCT group compared to the HLA-matched donor group (44.2% vs. 21.6%, p<0.001). Finally, the overall response rate of CMV-CTL was 89.7% (26/29 patients) in CMV infection after allo-HSCT. We concluded that CMV infection following allo-HSCT correlated with increased mortality in children’s patients, and haplo-HSCT was an independent risk factor for CMV infection. Adoptive CMV-CTL cell therapy was safe and effective in pediatric patients with CMV infection.

## Introduction

Cytomegalovirus (CMV) infection is a common and critical viral infection in allogeneic hematopoietic stem cell transplant (allo-HSCT) (Stern and Papanicolaou, 2019; Shafat et al., 2020). The incidence of CMV infection is around 40-70% based on diverse types of transplantation as well as territory (Walker et al., 2007; Cannon et al., 2010; Schmidt-Hieber et al., 2013; Dziedzic et al., 2017; Solano et al., 2021; Aristizabal et al., 2022). CMV infection is associated with an increased risk of mortality (Dziedzic et al., 2017; Stern and Papanicolaou, 2019) [hazard ratio of 1.5 from a systemic review (Gimenez et al., 2019)]. Antiviral medication is often used for pre-emptive treatment strategies, however, the incidence of CMV disease in CMV viremia is about 10% following allo-HSCT despite pre-emptive antiviral treatment. Subsequently, emerging reports indicated it was effective for the treatment of CMV-specific cytotoxic T lymphocytes (CMV-CTL) after allo-HSCT (O'Reilly et al., 2016; van der Heiden et al., 2018; Shafat et al., 2020).

In the current study, we used retrospective children’s cohorts to address the incidence and risk factors of CMV infection after allo-HSCT. In addition, we evaluated the safety and efficacy of donor-derived and third-party CMV-CTL in this population.

## Methods

### Patients

Between January 2017 and July 2021, 382 patients who underwent allo-HSCT from HLA-matched or mismatched relative or unrelative donors in the Department of Pediatrics, Nanfang Hospital, Southern Medical University were enrolled. The institutional review board at the hospital approved the protocol, and all consent forms approved by the institution were signed. The patients’ characteristics were shown in **Table 1**.

**Table 1:** Characteristics of patients by CMV infection.

	no CMV infection (n=255)	CMV infection (n=127)	P value
**Age (year, mean (SD))**	6.68 (3.38)	7.09 (4.06)	0.297
**Gender (%)**			
Male	165 (64.7)	86 (67.7)	0.639
Female	90 (35.3)	41 (32.3)	
**Diagnosis (%)**			
ALL	3 (1.2)	14 (11.0)	<0.001
AML	22 (8.6)	18 (14.2)	
SAA	19 (7.5)	11 (8.7)	
TM	169 (66.3)	63 (49.6)	
JMML	24 (9.4)	9 (7.1)	
PID	11 (4.3)	7 (5.5)	
Others	7 (2.7)	5 (3.9)	
**CMV serostatus, IgG (%)**			
R+/D+	146 (57.3)	70 (55.1)	0.790
R-/D+ or R+/D-	45 (17.6)	21 (16.5)	
Not available	64 (25.1)	36 (28.3)	
**Donor type (%)**			
Relative	169 (66.3)	91 (71.7)	0.344
Unrelative	86 (33.7)	36 (28.3)	
**HLA-typing (%)**			
Matched	157 (61.6)	48 (37.8)	<0.001
Haploidentical	98 (38.4)	79 (62.2)	
**CB engraftment (%)**			
No	201 (78.8)	78 (61.4)	<0.001
Yes	54 (21.2)	49 (38.6)	
**MNC (×10^8^/kg, median [IQR])**	8.15 [8.00, 26.81]	21.30 [8.00, 29.06]	0.003
**CD34 (×10^6^/kg, median [IQR])**	7.12 [4.00, 12.35]	8.29 [5.04, 13.12]	0.074
**Conditioning regimen (%)**			
Cy/Bu/Flu/TT	122 (47.8)	51 (40.2)	0.001
Cy/Bu/Flu/VP16	5 (2.0)	11 (8.7)	
Cy/Bu/Flu	106 (41.6)	50 (39.4)	
Cy/Bu/Flu/TBI	16 (6.3)	10 (7.9)	
Cy/Flu	5 (2.0)	0 (0.0)	
Cy/Bu/Flu/TT/VP16	1 (0.4)	5 (3.9)	
**Rituximab used in conditioning (%)**			0.619
No	206 (80.8)	106 (83.5)	
Yes	49 (19.2)	21 (16.5)	
**ATG used in conditioning (%)**			0.035
No	59 (23.1)	43 (33.9)	
Yes	196 (76.9)	84 (66.1)	
**PTCY used in conditioning (%)**			
No	151 (59.2)	50 (39.4)	<0.001
Yes	104 (40.8)	77 (60.6)	
**Prophylaxis of GVHD (%)**			<0.001
CsA/MMF	57 (22.4)	14 (11.0)	
Tacrolimus/MMF	67 (26.3)	71 (55.9)	
CsA/MMF/MTX	115 (45.1)	38 (29.9)	
Tacrolimus/MMF/MTX	16 (6.3)	4 (3.1)	
**Neutrophil recovery, days (median [IQR])**	20.00 [17.00, 23.75]	22.00 [16.00, 31.00]	0.020
**Hemoglobin recovery, days (median [IQR])**	16.00 [13.00, 23.00]	20.00 [13.00, 33.00]	0.021
**Platelet recovery, days (median [IQR])**	14.00 [12.00, 23.00]	18.50 [12.00, 35.50]	0.006
**Engraftment (%)**			0.257
Engrafted	241 (94.5)	124 (97.6)	
Graft failure	14 (5.5)	3 (2.4)	
**Acute GVHD, no. of patients (%)**			0.106
None	185 (72.5)	83 (65.4)	
Grade I-II	43 (16.9)	33 (26.0)	
Grade III-IV	27 (10.6)	11 (8.7)	
**Overall survival time (months, median [IQR])**	44.00 [25.50, 56.00]	36.00 [20.50, 49.50]	0.002
**Survival status (%)**			0.016
Alive	242 (94.9)	111 (87.4)	
Death	13 (5.1)	16 (12.6)	

ALL, acute lymphoblast leukemia; AML, acute myeloid leukemia; ATG, antithymocyte globulin; GVHD, graft-versus-host disease; Bu, busulfan; CB, cord blood; CMV, cytomegalovirus; CsA, cyclosporine A; Cy, cyclophosphamide; D, donor; Flu, fludarabine; JMML, juvenile myelomonocytic leukemia; MMF, mycophenolate mofetil; MNC, mononuclear cells; MTX, methotrexate; PID, primary immunodeficiency; PTCY, post-transplantation cyclophosphamide; R, recipient; SAA, severe aplastic anemia; TBI, total body irradiation; TM, thalassemia major; TT, thiotepa; VP16, etoposide.

### Transplant protocol

The donor selection and the transplant protocol were administered as previously reported (Li et al., 2012; Li et al., 2019; He et al., 2020; Xiao et al., 2021; Wen et al., 2022). In brief, a myeloablative conditioning regimen like busulfan (Bu)/cyclophosphamide (Cy)/fludarabine (Flu) with or without thiotepa (TT) was used for HLA-matched relative or unrelative donor transplant with anti-thymocyte globulin (ATG) as *in vivo* T cell depletion. As for HLA-haploidentical HSCT (haplo-HSCT), peripheral blood stem cell (PBSC) from a relative donor and cord blood (CB) from an unrelative donor were infused on day 0 and day 6 respectively using post-transplantation cyclophosphamide (PTCY) regimen.

### CMV monitoring, definitions, and antiviral therapy

Plasma CMV-DNA detection by qPCR was conducted weekly after allo-HSCT at least for 12 weeks. CMV infection was defined as the detection of viral nucleic acid in any body fluid or tissue specimen. CMV viremia was defined as the detection of CMV-DNA only in samples of plasma. Recurrent CMV infection was defined as new detection of CMV infection in a patient who had previously been diagnosed with CMV infection, and in whom the CMV turned negative for at least 4 weeks. According to previously published criteria CMV disease was diagnosed relying upon CMV nucleic acid detection (Ljungman et al., 2002). The drug resistance mutated genes of CMVwere not applicable in this study. Pre-emptive therapy was initiated with ganciclovir (5 mg/kg intravenously, twice daily) when a CMV infection was documented, and this treatment was continued with the combination of foscarnet (60 mg/kg intravenously, two to three times per day) or cidofovir (5 mg/kg intravenously, biweekly) when the CMV infection progressed. The CMV-CTL therapy was indicated for those who experienced refractory or recurrent CMV viremia and CMV diseases.

### Generation of CMV-CTL

Commercial CMV-CTL (iCell Biotechnology, Guangzhou, China) was applied in this study according to a previous publication (Pei et al., 2017). In brief, peripheral blood mononuclear cells were isolated from the donor. IFN-γ was added on day 0, and then IL-2 and anti-CD3 antibodies were added on day 1. After two times of antigen stimulation using the CMVpp65 peptide, CMV-CTL cells were selected depending on whether a sufficient number (≥10^7^) of CMV-specific T cells had been generated (Pei et al., 2017).

### Statistical analysis

Two-tailed t-tests or Kruskal-Wallis test were conducted for continuous variables between groups, while the chi-square test or Fisher’s exact test was for categorical variables. Logistic regression models for binary variables were used for both univariate and multivariate analyses (all factors in univariate analyses with a P value < 0.10 were included for multivariate analyses). The cumulative incidence of CMV infection was calculated by a competing risk model. The probability of OS was determined by the Kaplan-Meier method and compared with the log-rank test. P value < 0.05 was considered to indicate statistical significance. All analyses were performed using the R (version 4.2.1, available online at http://www.R-project.org).

## Results

### Patient characteristics and outcomes

A total of 382 children’s patients who underwent allo-HSCT were enrolled in this study, consisting of 255 patients without CMV infection and 127 patients with CMV infection. The patient characteristics and clinical outcomes were depicted in **Table 1**. 104 patients received matched sibling donors (MSDs) transplants and 101 patients with matched unrelated donors (MUDs) transplants. Moreover, haplo-HSCT was conducted for 177 patients. Thalassemia major (TM) accounted for 60.7% of the disease composition. Most patients received myeloablative conditioning, whereas only five patients received reduced intensive conditioning (Cy/Flu). There were significant differences between with and without CMV infections in terms of disease types, HLA-typing, engraftment of CB, the number of mononuclear cells (MNC) transfusions, conditioning regimens, graft versus host disease (GVHD) prophylaxis, and survival status **(** **Table 1** **)**.

**Figure 1:**
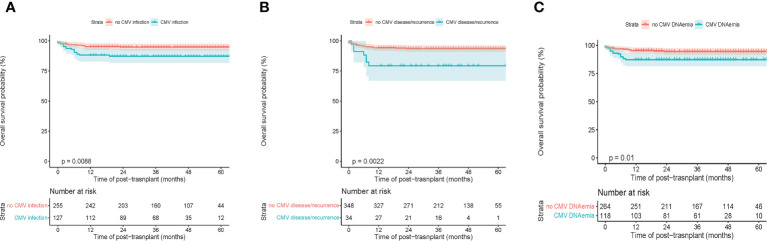
Transplant outcomes between subgroups based on CMV infection in children’s patients. **(A)** Overall survival (OS) between with and without CMV infection. **(B)** OS between with and without CMV disease or recurrent CMV. **(C)** OS between with or without CMV viremia.

In general, the 5-year overall survival (OS) of the whole population was 92.3% ± 1.4% [95% CI (89.7%-95.1%)] **(** **Supplemental Figure 1A** **)**. The OS of malignant diseases was significantly lower than nonmalignant diseases, 82.3% ± 3.8% [95% CI (75.2%-90.0%)] and 96.0% ± 1.2% [95% CI (93.7%-98.3%)], respectively (p<0.001) **(** **Supplemental Figure 1B** **)**. The rate of primary graft failure was 4.5%. Of note, CMV infection resulted in a significantly poorer 5-year OS of 87.3% ± 3.0% [95% CI (81.7%-93.3%)] compared to non-CMV infection 94.8% ± 1.4% [95% CI (92.1%-97.6%)] **(** **Figure 1A** **)**. However, there was no statistical difference in either nonmalignant or malignant disease subgroups comparison **(** **Supplemental Figure 2** **)**. Regarding the CMV disease, the 5-year OS between with or without CMV disease were 79.4% ± 6.9% [95% CI (66.0%-94.2%)] and 93.6% ± 1.3% [95% CI (91.0%-97.4%)], respectively (p=0.002) **(** **Figure 1B** **)**. Furthermore, the 5-year OS of CMV viremia and non-CMV viremia were 87.3 ± 3.1% [95% CI (81.5%-93.5%)] and 94.6 ± 1.4% [95% CI (91.9%-97.4%)], respectively (p=0.010) **(** **Figure 1C** **)**.

### Incidence of CMV infection

CMV viremia occurred in a total of 124 patients (32.5%) with a median time of 48 days after allo-HSCT. The median peak of CMV DNAemia was 3430 IU/mL. CMV disease was diagnosed in a total of 24 patients (6.3%), including 16 pneumonia, 4 enteritides, 3 cystitides, and 1 encephalitis. And the median duration of CMV DNAemia time was 20 days **(** **Table 2** **)**. Moreover, there were 63 cases of CMV infections (61 CMV viremia and 10 CMV disease). In TM patients, there were 25/64 (39.0%) haplo-HSCT patients with CMV infection while 38/168 (22.6%) in HLA-matched HSCT. In ALL and AML patients, the CMV infection rate in haplo-HSCT and HLA-matched HSCT were 27/47 (57.4%) and 5/10 (50.0%) respectively.

**Table 2:** Features of CMV infection.

	All patients (n=382)
**CMV DNAemia (%)**	
No	258 (67.5)
Yes	124 (32.5)
**Recurrent CMV infection (%)**	17 (100.0)
**CMV disease (%)**	
Pneumonia	16 (66.7)
Enteritis	4 (16.7)
Cystitis	3 (12.5)
Encephalitis	1 (4.2)
**CMV DNAemia time (days after allo-HSCT) (median [IQR])**	48.00 [37.75, 59.00]
**Peak of CMV DNA-PCR (IU/mL) (median [IQR])**	3430.00 [1762.50, 11475.00]
**Duration of CMV infection (days) (median [IQR])**	20.00 [13.00, 27.25]

**Figure 2:**
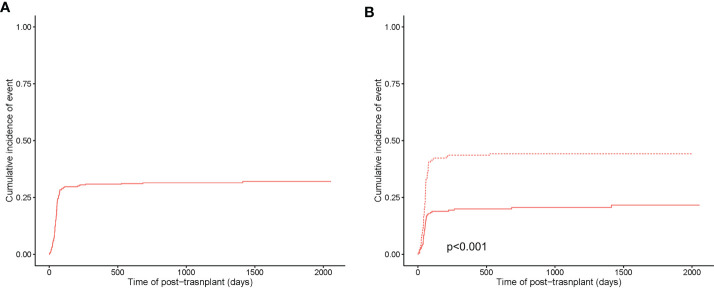
Cumulative incidence of CMV infection. **(A)** Cumulative incidence of CMV infection in the entire population. **(B)** Cumulative incidence of CMV infection between HLA-matched (in a solid curve) and haploidentical transplant (in a dashed curve).

The 5-year cumulative incidence of CMV infection was 32.1% ± 0.1% **(** **Figure 2A** **)**. In subgroup analysis, the 5-year cumulative incidence of CMV infection in the HLA-matched donor group was 21.6% ± 0.1%, while the haplo-HSCT group was 44.2% ± 0.1% (p<0.001) **(** **Figure 2B** **)**.

### Risk factor analyses for CMV infection after allo-HSCT

In univariate analysis **(** **Table 3** **)**, CMV infection more potentially occurred in ALL and AML than TM with P values of 0.028 and <0.001, respectively. The application of ATG in the conditioning regimen was a protective factor for CMV infection (P=0.026). However, the usage of VP16 in the conditioning regimen increased the risk of CMV infection compared to Cy/Bu/Flu/TT (P value of Cy/Bu/Flu/VP-16 was 0.003, P value of Cy/Bu/Flu/TT/VP-16 was 0.025). Interestingly, only grade I-II aGVHD was associated with a high incidence of CMV infection (P=0.023). Of note, haploidentical donors, using PTCY in conditioning regimen, UCB engraftment, and high MNC transfusion dramatically elevated the risk of CMV infection (P<0.001, P<0.001, P<0.001, P=0.007 respectively). Late recovery of neutrophils, hemoglobin, and platelet had a significant association with CMV infection (P=0.003, P=0.005, P=0.001, respectively).

**Table 3:** Univariate and multivariate analysis of risk factors affecting CMV infection.

	Univariate analysis		Multivariate analysis	
Factors	OR (95%CI)	P value	OR (95%CI)	P value
**Age**	1.032 (0.973-1.094)	0.296		
**Male vs. female**	0.874 (0.553-1.368)	0.559		
**Underlying disease**				
TM vs. AML	2.161 (1.079-4.289)	0.028	1.732 (0.434-7.085)	0.432
TM vs. ALL	11.442 (3.550-51.107)	<0.001	7.332 (1.406-47.475)	0.023
TM vs. SAA	1.529 (0.670-3.344)	0.296	1.422 (0.576-3.392)	0.433
TM vs. JMML	0.990 (0.416-2.178)	0.981	0.723 (0.140-3.635)	0.692
TM vs. PID	1.680 (0.595-4.458)	0.304	1.617 (0.418-5.968)	0.470
TM vs. others	1.886 (0.541-6.122)	0.293	1.254 (0.321-4.598)	0.734
**CMV serostatus (IgG)**				
R+/D+ vs. R+/D- or R-/D+	0.973 (0.531-1.742)	0.929		
R+/D+ vs. unknown	1.173 (0.710-1.925)	0.529		
**ATG not used vs. used**	0.588 (0.368-0.942)	0.026	1.527 (0.401-6.272)	0.540
**PTCY not used vs. used**	2.236 (1.452-3.469)	<0.001	0.994 (0.361-2.655)	0.990
**GVHD prophylaxis**				
CsA+MMF+MTX vs. CsA+MMF	3.207 (1.964-5.304)	<0.001		
CsA+MMF+MTX vs. Tacrolimus+MMF	0.743 (0.363-1.456)	0.400		
CsA+MMF+MTX vs. Tacrolimus+MMF+MTX	0.757 (0.207-2.214)	0.636		
**Engraftment vs. graft failure**	0.416 (0.095-1.305)	0.175		
**CB not used vs. CB used**	2.338 (1.466-3.735)	<0.001	1.125 (0.541-2.347)	0.753
**Relative donor vs. unrelative donor**	0.777 (0.484-1.231)	0.289		
**HLA-matched vs. haploidentical**	2.637 (1.707,4.108)	<0.001	2.649 (1.118-6.396)	0.027
**Rituximab not used vs. used**	0.833 (0.467,1.445)	0.524		
**Conditioning regimen**				
Cy/Bu/Flu/TT vs. Cy/Bu/Flu/VP16	5.263 (1.817-17.413)	0.003		
Cy/Bu/Flu/TT vs. Cy/Bu/Flu/TT/VP-16	11.961 (1.869-232.211)	0.025		
Cy/Bu/Flu/TT vs. Cy+Bu+Flu	1.128 (0.706-1.805)	0.614		
Cy/Bu/Flu/TT vs. Cy/Bu/Flu/TBI	1.495 (0.618-3.476)	0.357		
Cy/Bu/Flu/TT vs. Cy/Flu	–	0.982		
**MNC, ×10^8^/kg**	1.023 (1.006-1.040)	0.007	1.000 (0.967-1.033)	0.987
**CD34, ×10^6^/kg**	1.017 (0.989-1.045)	0.228		
**Neutrophil recovery, day**	1.041 (1.014-1.069)	0.003	1.015 (0.981-1.049)	0.391
**Platelet recovery, day**	1.026 (1.011-1.043)	0.001		
**Acute GVHD**				
**None vs. I-II**	1.711 (1.011-2.889)	0.044	2.027 (1.100-3.735)	0.023
**None vs. III-IV**	0.908 (0.414-1.872)	0.800	0.971 (0.399-2.218)	0.946

ATG, antithymocyte globulin; ALL, acute lymphoblast leukemia; AML, acute myeloid leukemia; GVHD, graft-versus-host disease; Bu, busulfan; CB, cord blood; CMV, cytomegalovirus; CsA, cyclosporine A; Cy, cyclophosphamide; D, donor; Flu, fludarabine; JMML, juvenile myelomonocytic leukemia; MMF, mycophenolate mofetil; MNC, mononuclear cells; MTX, methotrexate; OR, odd ratio; PID, primary immunodeficiency; PTCY, post-transplantation cyclophosphamide; R, recipient; SAA, severe aplastic anemia; TBI, total body irradiation; TM, thalassemia major; TT, thiotepa; VP16, etoposide.

Factors with a P value of <0.10 in univariate analysis were included in the subsequent multivariate analysis **(** **Table 3** **)**. In the multivariate analysis, it was intriguing that only ALL patients, haplo-HSCT, and grade I-II aGVHD were independent risk factors (P=0.023, P=0.027, P=0.023, respectively).

### Safety and efficacy of CMV-CTL

There were 29 patients with CMV infection who received CMV-CTL treatment, including 18 (62.1%) patients with CMV disease, 11 (37.9%) patients with CMV reoccurrence, and 8 (27.6%) patients with EBV reaction. The median time of transfusion of CMV-CTL was 2 (range 1-9). The median total nucleated cell (TNC) and CTL cell number of CMV-CTL was 0.18*10^9^/kg and 0.95*10^7^/kg, respectively. The therapy was effective in 26 (89.7%) patients. In detail, the overall response rate of donor-derived CMV-CTL and third-party CMV-CTL was 17/19 (89.5%) vs. 9/10 (90.0%), respectively. However, 3 (10.3%) patients did not respond to it. In addition, 3 out of 6 dead patients resulted from CMV progress. No acute allergic disease or hypotension was found during the transfusion of CMV-CTL. Moreover, no GVHD progress was not found in any patients after the therapy of CMV-CTL (**Table 4** and **Supplemental Table 1**).

**Table 4:** Features of patients using CMV-CTL.

	Overall (n=29)
**Gender (%)**	
Male	18 (62.1)
female	11 (37.9)
**CMV serostatus, IgG (%)**	
R+/D+	6 (20.7)
R-/D+ or R+/D-	13 (44.8)
Not available	10 (34.5)
**Donor type by HLA (%)**	
Matched	15 (51.7)
Haploidentical	14 (48.3)
**Stem cell resource (%)**	
PBSC	17 (58.6)
CB	12 (41.4)
**Conditioning (%)**	
Bu/Cy/Flu	9 (31.0)
Bu/Cy/Flu/TT	18 (62.1)
Cy/Flu/TT	1 (3.4)
Bu/Cy/Flu/TT/TBI	1 (3.4)
**GVHD prophylaxis (%)**	
CsA/MMF	6 (20.7)
Tacrolimus/MMF	16 (55.2)
CsA/MMF/MTX	7 (24.1)
**Grade of aGVHD (%)**	
No	12 (41.4)
II	4 (13.8)
III	12 (41.4)
IV	1 (3.4)
**Grade of cGVHD (%)**	
No	24 (82.8)
Moderate	2 (6.9)
Severe	3 (10.3)
**Found by day post-transplant, days (median [IQR])**	45.00 [34.00, 55.00]
**Peak of CMV-DNA, IU/mL (median [IQR])**	6290.00 [2710.00, 41300.00]
**CMV disease (%)**	
None	11 (37.9)
Pneumonia	14 (48.3)
Enteritis	4 (13.8)
**Recurrence of CMV (%)**	
No	18 (62.1)
Yes	11 (37.9)
**Duration of CMV infection, day (median [IQR])**	27.00 [12.00, 56.00]
**Times of CMV-CTL infusion (%)**	
1	6 (20.7)
2	18 (62.1)
4	4 (13.8)
9	1 (3.4)
**Resource of CMV-CTL (%)**	
Donor-derived	19 (65.5)
Third-party	10 (34.5)
**Quality control of CMV-CTL**	
TNC, *10^9^/kg (median [IQR])	0.18 [0.12, 0.24]
CTL, *10^7^/kg (median [IQR])	0.95 [0.64, 1.65]
Viability (median [IQR])	95.90 [95.20, 97.25]
CD3+CD4+, % (median [IQR])	4.40 [2.58, 5.20]
CD3+CD8+, % (median [IQR])	95.10 [92.45, 96.30]
CD8+NKG2D+, % (median [IQR])	92.70 [88.05, 94.30]
IFN-γ+, % (median [IQR])	5.75 [3.20, 11.80]
**Overall response (%)**	
No response	3 (10.3)
Response	26 (89.7)
**Survival status (%)**	
Alive	23 (79.3)
Dead	6 (20.7)

aGVHD, acute graft-versus-host disease; Bu, busulfan; CB, cord blood; cGVHD, chronic graft-versus-host disease; CMV, cytomegalovirus; CsA, cyclosporine A; CTL, cytotoxic t lymphocyte; Cy, cyclophosphamide; D, donor; Flu, fludarabine; MMF, mycophenolate mofetil; MTX, methotrexate; PBSC, peripheral blood stem cell; PGF, primary graft failure; R, recipient; TBI, total body irradiation; TNC, total nucleated cell; TT, thiotepa.

## Discussion

This study included a relatively large cohort of TM patients; thus, it is a representative study regarding CMV infection in TM. Accordingly, a recent study showed that 15 out of 20 (75%) CMV reactivation was found in PTCY-based haploidentical HSCT for TM (Vellaichamy Swaminathan et al., 2021). Regarding matched sibling donor HSCT using *in vivo* T-cell depletion myeloablative conditioning, the rate of CMV reactivation was 36.2% (Qin et al., 2019). Notably, we reported that a higher incidence of CMV infection was found in ALL and AML compared to TM patients. The potential reasons included that more haplo-HSCTs were performed and more CB engraftments were found in leukemia patients compared to TM patients based on our transplant regimen (Xiao et al., 2021). Our result was quite similar to a current study in which the cumulative incidences of CMV DNAemia in the MSD, MUD and haploidentical groups were 39.0, 55.6, and 85.7%, respectively (Lin et al., 2019). Another retrospective pediatric patient study from a single center in Latin America showed that the cumulative incidence of CMV viremia was 70.5% while CMV disease was 4.7% (Aristizabal et al., 2022).

There were a series of studies that reported that CMV reactivation resulted in a less relapsed rate in leukemia patients potentially associated with effect T cell activation (Takenaka et al., 2015; Inagaki et al., 2016; Yokoyama et al., 2020). Meanwhile, this benefit was nullified by the increased nonrelapse mortality. In our study, there was no difference in survival analysis of CMV infection in both nonmalignant disease and malignant disease.

The main finding in our study was that haplo-HSCT increased the cumulative incidence of CMV infection based on both univariate and multivariate analysis. In univariate analysis, only using ATG was a protective factor while using PTCY, applying UCB, and higher MNC were risk factors. These are mainly caused by our haplo-HSCT administration using the PTCY regimen instead of ATG. High MNC of PBSC from relative donors combined with unrelated CB were applied. And PTCY with or without a low dose of ATG rather than a regular dose of ATG *in vivo* T cell depletion in haplo-HSCT in our study (Li et al., 2019; Xiao et al., 2021). Consistently, a series of studies support CMV infection occurred more frequently in haplo-HSCT due to delayed T-cell reconstitution (Dziedzic et al., 2017; Lin et al., 2019; Yokoyama et al., 2019; Solano et al., 2021; Cho et al., 2021). In most studies (Kang et al., 2021; Wang et al., 2022), ATG seems like a risk factor. For example, significantly more CMV reactivation rate 70.3% was found in high dose of ATG (unrelated: 7.5 mg/kg, haploidentical: 10.0 mg/kg) than low dose (unrelated: 3.75 mg/kg, haploidentical: 5.0 mg/kg) 51.3% in leukemia children (Kang et al., 2021). On the other hand, a multicenter study showed that the incidence of CMV viremia was lower in non-T-cell depleted haplo-HSCT ALL patients (Nagler et al., 2021). An interesting study found that the administration of ATG abrogated relapse protection following CMV reactivation in AML patients which we previously discussed (Turki et al., 2022). In our study, only I-II aGVHD was linked to a significantly high incidence of CMV infection in univariate analysis. However, there was no statistical difference in the III-IV aGVHD subgroup which may result from the relatively lower incidence of III-IV aGVHD incidence in our study. Overall, the I-IV aGVHD more occurred in the CMV infection group (**Table 1**). Similarly, aGVHD required intensive immunosuppression and CBT comes with delayed immune system reconstitution, therefore major studies identified both aGVHD and CBT as risk factors for CMV infection (Walker et al., 2007; Takenaka et al., 2015; Yokoyama et al., 2020; Zhu et al., 2021; Akahoshi et al., 2022). Unlike other studies (Ganepola et al., 2007; Schmidt-Hieber et al., 2013; Takenaka et al., 2015), we have not identified the CMV serostatus of donor and recipient as a significant factor, which might result from the existing unknown data. Eventually, it was well established that CMV infection was associated with overall mortality especially CMV disease (Rowe et al., 2016; Aristizabal et al., 2022; Ru et al., 2022). Therefore, we recommend that it was required to carefully and rigorously monitor CMV infection after haplo-HSCT in leukemia patients in whom letermovir may be appropriately used for prophylaxis in the future study (2).

In addition, we herein reported that a small cohort of children’s patients received CMV-CTL therapy, which was safe, effective, and well tolerated. Theoretically, adoptive immunotherapy of CMV-CTL can reduce the risk of CMV infection and subsequently restore the CMV T cell immunity after HSCT in about 80% of patients (2).

CMVpp65-specific donor-derived CTL, a most common type of CMV-CTL used in current clinical trials, applied for preemptive therapy along with antiviral therapy in haplo-HSCT recipients reduced the risk of persistent and late CMV infection and improved 1-year overall survival compared to matched controls (Zhao et al., 2020). An alternative approach to donor-derived CTL was third-party CMV-CTL, especially for those who underwent MUD-HSCT or CBT. Unlike donor-derived CMV-CTL which may persist for up to 10 years, third-party CMV-CTL only sustained only up to 3 months (2). Therefore, it required multiple infusions to maintain the CMV-CTL therapeutic effect (O'Reilly et al., 2016). Moreover, a recent report noted that both third-party and donor-derived CMV-CTL triggered comparable antiviral responses to CMV infection through the restoration of endogenous CMV-specific immunity (Pei et al., 2022). Similar results were found in our study. According to the overall response rate of 70-100% in a literature review(1), it was 89.7% in our report.

Nevertheless, the conclusion of our study was restricted by inevitable limitations, including the common drawbacks of a single-center retrospective study, the diversity of graft sources, conditioning regimens and methods of *in vivo* T cell depletion, deviations in treatments of complications, and insufficiency of representativeness. Furthermore, immune reconstitution in CMV infection patients should also be investigated in the future.

In conclusion, CMV infection following allo-HSCT correlated with poor OS in children’s patients. Haplo-HSCT was an independent risk factor for CMV infection. Adoptive CMV-CTL cell therapy was safe and effective in children’s patients with CMV infection.

## Data availability statement

The original contributions presented in the study are included in the article/**Supplementary Material**. Further inquiries can be directed to the corresponding authors.

## Ethics statement

The studies involving human participants were reviewed and approved by Ethics Committee of Nanfang Hospital of Southern Medical University. Written informed consent to participate in this study was provided by the participants’ legal guardian/next of kin.

## Author contributions

YR conceptualized the study. YR, TL, and QL collected, analyzed, and interpreted clinical data. YR and TL wrote the original draft. YR, TL, QL, XL, LC, JW, YX, DX, YH, XW, and XF wrote and edited the manuscript. YR and XF supervised the study and YR acquired funding. All authors have contributed to the manuscript and approved the submitted version.

## Funding

This work was supported by the GuangDong Basic and Applied Basic Research Foundation (Grant No.2022A1515010012), and the President Foundation of Nanfang Hospital, Southern Medical University (Grant No. 2020C003).

## Conflict of interest

The authors declare that the research was conducted in the absence of any commercial or financial relationships that could be construed as a potential conflict of interest.

## Publisher’s note

All claims expressed in this article are solely those of the authors and do not necessarily represent those of their affiliated organizations, or those of the publisher, the editors and the reviewers. Any product that may be evaluated in this article, or claim that may be made by its manufacturer, is not guaranteed or endorsed by the publisher.

## Supplementary material

Supplementary Figure 1
**(A)** Overall survival (OS) of the entire population. **(B)** OS between nonmalignant diseases and malignant diseases.

Supplementary Figure 2
**(A)** Overall survival (OS) between CMV infection in nonmalignant diseases. **(B)** OS between CMV infection in malignant diseases.
