# Supplementary figures and images for: Features of cytomegalovirus infection and evaluation of cytomegalovirus-specific T cells therapy in children’s patients following allogeneic hematopoietic stem cell transplantation: A retrospective single-center study
Source: Front Cell Infect Microbiol. 2022 Oct 20;12:1027341. doi: 10.3389/fcimb.2022.1027341 (PMC9630835; doi:10.3389/fcimb.2022.1027341)

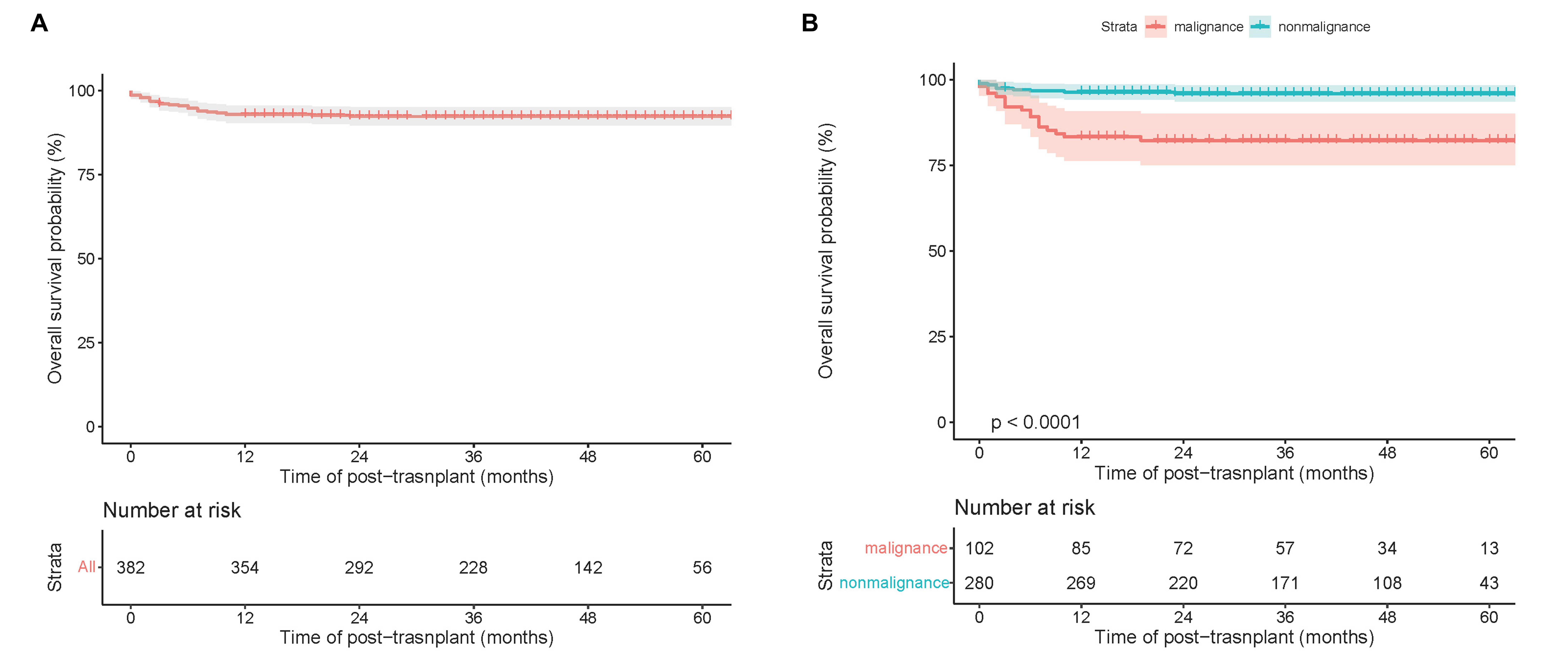

Supplement: Supplementary Figure 1 — (A) Overall survival (OS) of the entire population. (B) OS between nonmalignant diseases and malignant diseases. [file Image_1.jpeg]

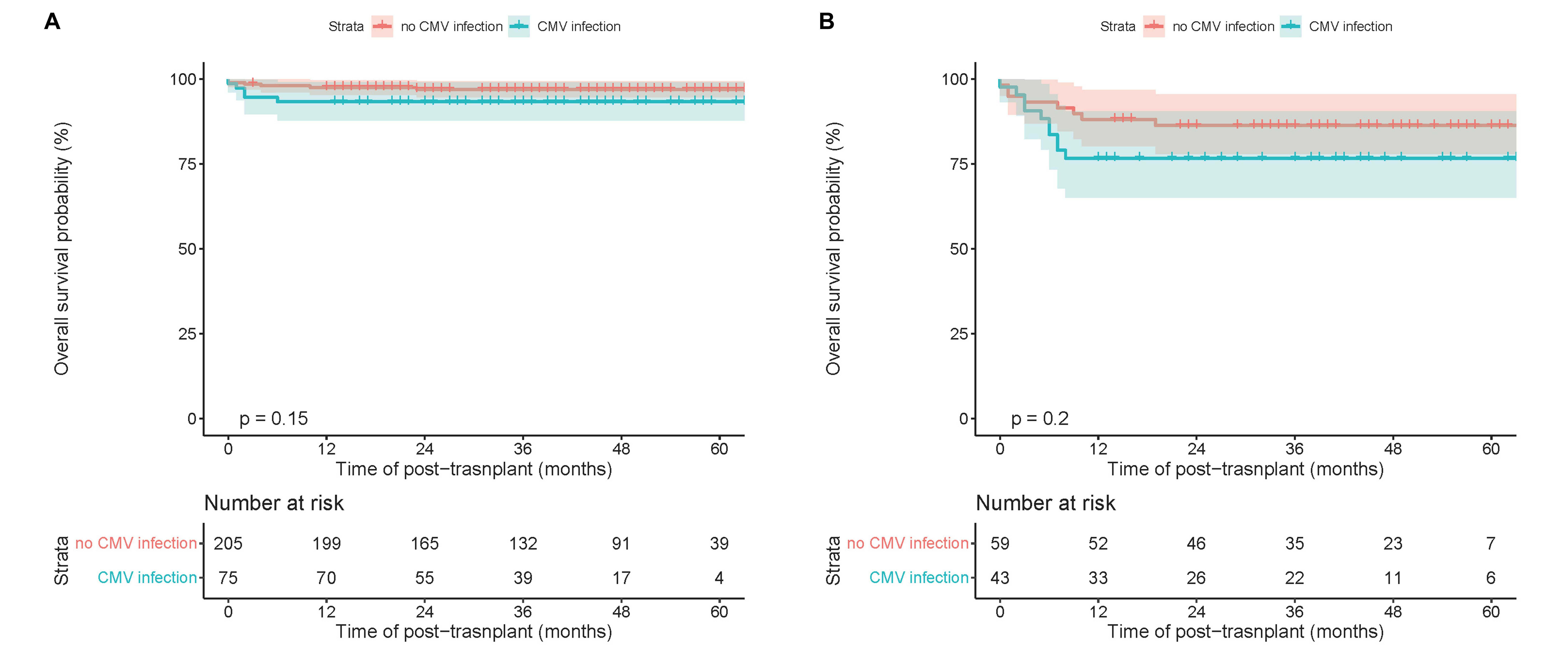

Supplement: Supplementary Figure 2 — (A) Overall survival (OS) between CMV infection in nonmalignant diseases. (B) OS between CMV infection in malignant diseases. [file Image_2.jpeg]
